# Supplementary material for: Pyruvate Dehydrogenase Kinase 1 inhibition mediated oxidative phosphorylation enhancement in cartilage promotes osteoarthritis progression
Source: BMC Musculoskelet Disord. 2023 Jul 20;24:597. doi: 10.1186/s12891-023-06585-6 (PMC10357736; doi:10.1186/s12891-023-06585-6)
Supplement: Supplementary file 1 — Additional file 1. [file 12891_2023_6585_MOESM1_ESM.zip › KEGG.Gsea.1653622432464/KEGG_RNA_POLYMERASE.html]

Details for gene set KEGG\_RNA\_POLYMERASE[GSEA]

|  || Dataset | 55457\_collapsed\_to\_symbols.cls.cls #OA\_versus\_normal.cls.cls #OA\_versus\_normal\_repos |
| Phenotype | cls.cls#OA\_versus\_normal\_repos |
| Upregulated in class | OA |
| GeneSet | KEGG\_RNA\_POLYMERASE |
| Enrichment Score (ES) | 0.35862085 |
| Normalized Enrichment Score (NES) | 1.192416 |
| Nominal p-value | 0.2063882 |
| FDR q-value | 0.3423883 |
| FWER p-Value | 1.0 |
Table: GSEA Results Summary

  

Fig 1: Enrichment plot: KEGG\_RNA\_POLYMERASE      
 Profile of the Running ES Score & Positions of GeneSet Members on the Rank Ordered List

  

| SYMBOL | TITLE | RANK IN GENE LIST | RANK METRIC SCORE | RUNNING ES | CORE ENRICHMENT || 1 | POLR2G | RNA polymerase II subunit G [Source:HGNC Symbol;Acc:HGNC:9194] | 183 | 0.830 | 0.0700 | Yes |
| 2 | POLR2J | RNA polymerase II subunit J [Source:HGNC Symbol;Acc:HGNC:9197] | 259 | 0.779 | 0.1433 | Yes |
| 3 | POLR2L | "RNA polymerase II, I and III subunit L [Source:HGNC Symbol;Acc:HGNC:9199]" | 333 | 0.733 | 0.2121 | Yes |
| 4 | POLR2I | RNA polymerase II subunit I [Source:HGNC Symbol;Acc:HGNC:9196] | 640 | 0.609 | 0.2501 | Yes |
| 5 | POLR3K | RNA polymerase III subunit K [Source:HGNC Symbol;Acc:HGNC:14121] | 651 | 0.605 | 0.3108 | Yes |
| 6 | POLR2K | "RNA polymerase II, I and III subunit K [Source:HGNC Symbol;Acc:HGNC:9198]" | 1090 | 0.499 | 0.3275 | Yes |
| 7 | POLR3C | RNA polymerase III subunit C [Source:HGNC Symbol;Acc:HGNC:30076] | 1504 | 0.429 | 0.3389 | Yes |
| 8 | POLR2E | "RNA polymerase II, I and III subunit E [Source:HGNC Symbol;Acc:HGNC:9192]" | 1760 | 0.389 | 0.3586 | Yes |
| 9 | POLR3B | RNA polymerase III subunit B [Source:HGNC Symbol;Acc:HGNC:30348] | 2220 | 0.336 | 0.3571 | No |
| 10 | POLR2C | RNA polymerase II subunit C [Source:HGNC Symbol;Acc:HGNC:9189] | 2882 | 0.262 | 0.3324 | No |
| 11 | POLR2B | RNA polymerase II subunit B [Source:HGNC Symbol;Acc:HGNC:9188] | 6426 | -0.037 | 0.0611 | No |
| 12 | POLR2A | RNA polymerase II subunit A [Source:HGNC Symbol;Acc:HGNC:9187] | 7103 | -0.088 | 0.0176 | No |
| 13 | POLR1D | RNA polymerase I and III subunit D [Source:HGNC Symbol;Acc:HGNC:20422] | 7611 | -0.128 | -0.0088 | No |
| 14 | POLR2D | RNA polymerase II subunit D [Source:HGNC Symbol;Acc:HGNC:9191] | 7726 | -0.136 | -0.0039 | No |
| 15 | POLR3F | RNA polymerase III subunit F [Source:HGNC Symbol;Acc:HGNC:15763] | 9229 | -0.250 | -0.0951 | No |
| 16 | POLR2F | "RNA polymerase II, I and III subunit F [Source:HGNC Symbol;Acc:HGNC:9193]" | 9352 | -0.259 | -0.0782 | No |
| 17 | POLR1C | RNA polymerase I and III subunit C [Source:HGNC Symbol;Acc:HGNC:20194] | 9405 | -0.265 | -0.0554 | No |
| 18 | POLR3G | RNA polymerase III subunit G [Source:HGNC Symbol;Acc:HGNC:30075] | 10018 | -0.323 | -0.0701 | No |
| 19 | POLR1E | RNA polymerase I subunit E [Source:HGNC Symbol;Acc:HGNC:17631] | 11274 | -0.468 | -0.1201 | No |
| 20 | POLR2H | "RNA polymerase II, I and III subunit H [Source:HGNC Symbol;Acc:HGNC:9195]" | 12167 | -0.635 | -0.1248 | No |
| 21 | POLR3D | RNA polymerase III subunit D [Source:HGNC Symbol;Acc:HGNC:1080] | 12490 | -0.744 | -0.0743 | No |
| 22 | POLR1B | RNA polymerase I subunit B [Source:HGNC Symbol;Acc:HGNC:20454] | 12841 | -1.045 | 0.0046 | No |
Table: GSEA details [plain text format]

  

Fig 2: KEGG\_RNA\_POLYMERASE      
 Blue-Pink O' Gram in the Space of the Analyzed GeneSet

  

Fig 3: KEGG\_RNA\_POLYMERASE: Random ES distribution      
 Gene set null distribution of ES for **KEGG\_RNA\_POLYMERASE**
